# Speech and Language Therapists’ Views Regarding Their Scope of Practice in Written Language

**DOI:** 10.1111/1460-6984.70313

**Published:** 2026-08-14

**Authors:** Carol Moxam, Claire d'Urban‐Jackson, Lauren Ackerman

**Affiliations:** ^1^ Newcastle University King Newcastle‐upon‐Tyne Tyne and Wear UK

**Keywords:** literacy, position statement, scope of practice, speech and language therapists, written language

## Abstract

**Background:**

Spoken and written language are closely linked, and Speech and Language Therapists (SLTs) have expertise in the language skills that support both. However, unlike countries such as the US and Australia, UK SLTs lack clear guidance on their role in written language. This study explores the views of SLTs working with adults (aged 16+) and children to inform consideration of a UK position statement.

**Aims:**

To examine UK SLTs’ views on their role and scope of practice in written language assessment and intervention, and to ascertain whether a position statement is needed.

**Methods and Procedures:**

In 2021, an online survey was completed by 511 SLTs across the UK. The survey explored SLTs’ views on their role in written language, alongside their reported involvement in direct and indirect written language assessment and intervention. Quantitative data was analysed using R (R Core Team, 2024).

**Outcomes and Results:**

SLTs working with adults or across adults and children are more likely than those working solely with children to report having knowledge, skills, and a role in written language. Those already working in this area held a more positive view of their role. Most UK SLT respondents (89%) supported the development of a UK position statement. There was also strong support for greater emphasis on the links between language and literacy in university training.

**Conclusions and Implications:**

SLTs working across the lifespan are more likely to engage in written language than those working only with children. Findings indicate a clear need for UK specific guidance on SLTs’ role in written language. Addressing this will require enhanced pre‐registration education and continuing professional development on the links between language and literacy.

**WHAT THE PAPER ADDS:**

*What is already known on the subject*

- Language is the foundation for reading (word reading and reading comprehension) and underpins spelling and writing skills. SLT training covers areas of language that are important for literacy development. Internationally, SLTs in countries such as the USA and Australia have clearer guidance and more established roles in supporting written language and literacy, whereas in the UK this guidance remains limited.

*What does this study add to existing knowledge*

- The paper enhances our understanding of UK SLTs’ views on their role in written language across the lifespan. It highlights differing views across those who work with adults and those who work with children, and their scope of practice within written language. The level of certainty around SLTs’ role is influenced by whether they work on written language, resource allocation, and/or training.

*What are the potential clinical implications of this work?*

- Key clinical implications include: greater clarity regarding the role and scope of practice of UK SLTs with written language across the lifespan; UK policy and guidance through, for example, a formal position statement; research into how current training institutions embed the links between language and literacy within their curricula; enhanced pre‐registration training and continuing professional development opportunities for practising clinicians; and a strategic approach to supporting therapists working with children through interprofessional education and education‐based placements.

## 1 Introduction

A growing body of research (e.g., Adlof and Hogan 2018; Brookshire et al. 2014; Moxam 2020; Scarborough 2001; Sedita 2023; Snow 2016, 2021) highlights the strong links between spoken and written language. Further, written language relies on spoken language, with Moss and Washbrook (2016) demonstrating that early spoken language skills predict literacy outcomes at age 11. Language and literacy are key predictors of educational and future outcomes (Education Endowment Foundation 2021) as literacy is now recognised as a foundational skill for academic achievement and success because of its critical role in supporting the expression of spoken and written thoughts and knowledge.

Many countries recognise SLTs’ scope of practice in written language assessment and intervention, including the USA (American Speech, Language Hearing Association: ASHA), Canada (Speech, Language and Audiology Canada: SAC), Australia (Speech Pathology Australia: SPA), South Africa (South Africa Speech, Language, Hearing Association: SASLHA) and New Zealand (New Zealand Speech Therapists’ Association: NZSTA). The United Kingdom's (UK) guidance on SLTs’ involvement in written language is less explicitly defined. This is at odds with the international stance and the literature demonstrating the link between spoken and written language and SLTs’ associated expertise. The argument for clarifying UK SLTs’ scope of practice in written language is compelling and may additionally support the advancement of the profession within the UK. Clarifying the scope of practice of SLTs could advance the profession in the UK through strengthening professional identity; improving interprofessional opportunities for collaboration; informing public and professional perceptions and value of the role; and supporting consistency in service delivery across the lifespan (RCSLT 2024). Reading and writing remain a relatively underexplored area of practice for SLTs in the UK, especially given that the issue of SLTs’ role in written language has a long history, particularly for those working with children (Apel 2002; Nelson et al. 2001; Snow et al. 2019; Spracher 2000). This raises the question of why the issue persists. The literature (Blood et al. 2010; Bridges and Kelley 2023; Serry and Levickis 2020; Stephenson et al. 2024, 2025; Watson et al. 2020) identifies several contributing factors, including lack of clarity of role, service model constraints, preservice training, unclear role boundaries, preparedness, and self‐efficacy. The recent attention within the UK on the role of SLTs in written language may reflect growing recognition of the intrinsic link between language and literacy (Adlof and Hogan 2018; Moxam 2020; Scarborough 2001; Stephenson et al. 2024). This recognition has led to professionals working in language and/or literacy becoming increasingly aware of, and acknowledging more explicitly, the relationship between spoken and written language (Moxam 2020; Scarborough 2001; Stephenson et al. 2024) and its relevance for practice (Watson et al. 2020; Yi and Erickson 2024). At the same time persisting challenges remain around practitioner self‐efficacy belief, clarity of role, service models, and preservice preparation, (Blood et al. 2010; Bridges and Kelley 2023; Serry and Levickis 2020). This has inevitably raised the issue of SLTs’ involvement in written language and its implications for the therapist and the profession more widely. Clarifying SLTs’ role in this area seems the best next step. To begin to address this issue, this paper explores SLTs’ views on their role in written language across the lifespan.

## 2 The Links Between Language and Literacy

Previous researchers have consistently demonstrated that spoken language skills are foundational to successful literacy development (Werfel et al., 2019; Snow 2021; Werfel et al. 2019). Both spoken and written language are linked because they share the same linguistic sub‐domains: phonology, morphology, semantics, syntax and pragmatics. Building on this integrated view, we position UK SLTs as having both an explicit role (what they do in practice) and an explicit remit (what the profession formally recognises) in written as well as spoken language. The symbiotic relationship between language and literacy reinforces the need for both to be supported within practice. The shared foundations of written and spoken language will be explored in the following sections.

### 2.1 Phonological Awareness

Phonological awareness is the ability to identify, reflect on and manipulate the sound structures of a language (Masterson and Apel 2000). As a core component of spoken language, it provides a shared foundation for literacy because sensitivity to sounds supports spelling, reading and writing development (Goswami and Bryant 1990; ASHA 2001). Research demonstrates that a solid foundation in phonological awareness supports and improves outcomes for spelling, reading and writing skills (Goswami and Bryant 1990; ASHA 2001). For example, the ability to segment and manipulate sounds and syllables in words is essential for spelling, while blending sounds supports reading words. Because phonological awareness underpins both spoken and written language, work targeting phonological skills has the potential to support written language development.

### 2.2 Morphology

Morphology is the study of how words are built and encompasses units of meaning including roots (e.g., act), inflections (e.g., acted, acting, acts) and derivations (e.g., action, active). Because English is a morphophonemic writing system (Apel et al. 2011) spelling encodes both phonological and morphological information. In spoken language, morphemes may vary in their realisation depending on the surrounding sounds. For example, the regular past‐tense *‐ed* has phonologically conditioned allomorphs (/t/, /d/, /ɪd/). In contrast, in written language this morpheme is consistently represented as *ed*. For example, walked, played, and wanted all share the same ‐ed ending despite differing pronunciations of /t/, /d/, and /ɪd/ respectively.

Morphological awareness (the ability to recognise and manipulate these units) supports spoken language through vocabulary growth and marking tense, number, and possession (Moxam 2020; Nagy et al. 2014). Strong morphological awareness also supports written language and reading comprehension (Bourassa et al. 2006; Goodwin and Ahn 2013; Moxam 2020). Evidence demonstrates that explicit morphological instruction within the written domain improves reading, spelling and written composition, including for those with developmental language disorders (Apel and Werfel 2014; Calder et al. 2018). Because morphology underpins both spoken and written language, with each reinforcing the other, SLTs working within this domain may be well positioned to support written language development.

### 2.3 Semantics

Lexical semantic knowledge refers to the understanding of word meanings and word relationships, which then drive listening comprehension, reading comprehension, spelling and writing (Moxam 2020; Sedita 2023; Wolf et al. 2019). Because spoken and written language share the same underlying semantic system, gains in vocabulary directly support literacy development, while actively engaging with print further extends lexical semantic knowledge (Nation and Snowling 2004). Lexical semantic knowledge may support learners in distinguishing homophones (e.g., *their, there, they're)* and homographs (e.g., *bear* as in the animal and *bear* as in to tolerate; *rose* as in *a red rose* and *rose* as in *the sun rose*), highlighting cross modal processing demands. These reciprocal links position semantics as a core domain of SLT expertise, providing a rationale for their involvement in supporting written language development (Apel and Lawrence 2011; Sedita 2023; Wolf et al. 2019).

### 2.4 Syntax

Syntax refers to the system used to organise words into sentences, shaping how we construct meaning in both spoken and written language. Syntactic knowledge concerns understanding how words combine to form coherent sentences. When applied as a skill it supports spoken and written expression. Written texts often contain more complex syntax than everyday conversation giving learners access to structures not typically spoken in language, thereby extending their syntactic experiences across the lifespan (Nation & Dawson, 2022). Sentence level skills such as clause structure and grammatical relationships contribute to reading comprehension beyond word recognition (Oakhill & Cain, 2012). Meta‐analytic evidence shows strong links between syntactic skill and reading comprehension across ages and language (Tong et al. 2025). Evidence from explicit grammar interventions (e.g., Shape Coding, MetaTaal) further demonstrates that strengthening syntax supports both spoken and written language performance (Balthazar et al., 2020).

### 2.5 Pragmatics

Pragmatic competence underpins both listening and reading comprehension because meaning often depends on context and inference beyond words (Taguchi 2024). The same language‐based cues used to interpret intent in conversation are required for inferring implied meanings, understanding figurative language, and maintaining coherence in texts (Oakhill et al. 2015). SLTs play an essential role across the lifespan, strengthening pragmatic skills that support spoken and written language, with gains in one linguistic sub‐domain reinforcing the other (ASHA 2001).

Building on the earlier discussion, Snow (2020) argues that SLTs’ deep knowledge and understanding of language domains that underpin written language places them in a uniquely strong position to advise on assessment and instructional approaches that effectively support reading, spelling and writing development. The increasingly evident links between language and literacy have important implications for SLTs working with children with speech, language and communication needs (SLCN). Research and practice consistently reinforce these connections (Duke and Cartwright 2021; Scarborough 2001; Sedita 2023), underscoring the importance of SLTs’ role in supporting written language. However, SLTs often feel they do not have the training (Stephenson et al. 2025) or necessary skills (Loveall et al. 2022) to provide written language intervention to children with written language differences. For adults (including adolescents aged 16+ who can legally consent to intervention in the UK), written communication is key for daily functional participation in everyday life, for example, emailing, texting, reading letters or cards or managing appointments. Acquired brain injury, such as a stroke or traumatic brain injury, can impair both spoken and written language (Brookshire et al. 2014). Research shows that 68% of people with aphasia experience reading differences (Brookshire et al. 2014). SLTs who work with adults commonly make use of assessment tools, such as Psycholinguistic Assessments of Language Processing in Aphasia (PALPA) (Kay et al. 1992), to assess written language difficulties that are a common feature of aphasia (Knollman‐Porter et al. 2015; Purdy et al. 2019). Those who work with adults play a key role in helping these individuals regain, adapt, or facilitate their communication skills, including reading and writing, which are vital for independence and quality of life (Webster et al. 2020).

Despite strong evidence linking spoken and written language and international recognition of SLTs’ role in literacy, uncertainty persists within the UK regarding SLTs’ scope of practice in written language across the lifespan. This lack of clarity highlights the need to explore UK SLTs’ views on their role and responsibilities in written language assessment and intervention.

### 2.6 Aims of Study

The purpose of this study was to explore UK SLTs’ views on their role and scope of practice in written language assessment and intervention. The motivation for this study was to gauge practitioner views on whether there is a need for a position statement in this professional community regarding the role and scope of practice for UK SLTs in written language.

The following research questions were addressed in this study:

- To what extent do SLTs perceive themselves as having a professional role in supporting written language development?
- How do SLTs view their involvement in written language domains?
- What are SLTs’ views on their knowledge and skills relating to written language domains?
- What are SLTs’ views on the scope and content of preregistration training necessary to prepare for working in written language domains?
- What do SLTs identify as key facilitators in their ability to work effectively with written language?
- To what extent do SLTs perceive that the profession would benefit from a position paper to clarify and support their role in written language?
- Is there a difference in SLTs’ perception of their role in written language based on the client population they work with?

## 3 Methods

We conducted a survey within the UK to explore SLTs’ views on their role in written language, including direct (e.g., face‐to‐face intervention or assessment) and indirect (e.g., advice, training, consultation) intervention.

### 3.1 Research Design

This study adopted a questionnaire‐based survey design using the Joint Information Systems Committee (JISC) online platform (Version Two) to elicit SLTs’ views on their role in written language. The survey targeted qualified SLTs of any grade or place of employment in England, Scotland, Wales and Northern Ireland.

### 3.2 Ethics Approval

Approval for the project was granted by a Research Ethics Committee at Newcastle University in March 2021.

### 3.3 Recruitment of Participants

Eligibility for participation in the study required individuals to be qualified and currently practising as UK SLTs. To achieve a sufficiently representative sample of SLTs working within the National Health Service (NHS), education and independent practice, several avenues were utilised to facilitate recruitment and dissemination of the survey. To this end, the survey link was disseminated through various channels, including the Royal College of Speech and Language Therapists (RCSLT), its electronic research newsletter, relevant Clinical Excellence Networks, work colleagues, a webinar presentation in January 2022, the Association of SLTs in Independent Practice, NHS trusts and charities such as National Association of Professional concerned with Language Impairment in Children (NAPLIC), Integrated Children's Additional Needs Service (ICAN; now Speech and Language UK) and Association Of All Speech Impaired Children (AFASIC). A reminder was sent to all those contacted at six months post initial dissemination and again a month before the closing of the survey. E‐mailed follow‐up reminders were sent out to increase the response rates. Responses were anonymous. The survey was open between September 2021 and August 2022. Respondents were required to complete the survey in one sitting.

### 3.4 Survey Design and Content

An online self‐completion survey questionnaire for SLTs was designed by the authors and piloted specifically for this study. Draft survey items were informed by information obtained from an SLT focus group comprising of four SLTs (3 SLTs working with children and one working with adults) and two educators and an education focus group comprising of a Special Educational Needs Co‐ordinator (SENCo), a class teacher and a Learning Support Assistant and a parent interview. In addition, a review of the relevant literature informed survey development. The survey items were subsequently refined through consultation with practising SLTs and academic researchers. Draft versions of the survey were modified in response to feedback from the focus groups, expert consultation and the literature until a final version was agreed upon by the authors.

The final survey contained 41 items which covered the following five sections: i) demographic characteristics, ii) SLTs’ views regarding their role in written language, iii) SLTs’ views on service delivery options for written language; iv) scope of practice; and v) perceived barriers and facilitators in working with written language. In the survey, respondents were asked about their skills and knowledge to work with written language; throughout the paper, ‘work with written language’ is used to denote professional activity focused on written language as an area of practice. For the purposes of this study, written language was conceptualised as comprising 12 areas: alphabetic knowledge/phonics (e.g., sounds represented by a letter or group of letters); phonological awareness; vocabulary; morphological awareness; word reading; sentence‐level reading; spelling; sentence‐level writing; written story narrative; reading comprehension; social/functional written skills (e.g., texting, emailing, functional communication such as writing job applications); and assistive technology for literacy (e.g., speech‐to‐text and text‐to‐speech).

The online survey provided a statement of consent on the first few pages. Response formats included yes/no questions, multiple choice, Likert scales, open ended questions, and opportunities throughout for respondents to provide free text answers to provide additional comments to set questions. Completion time was estimated at 15 minutes. All answers to the questionnaires were stored directly in the JISC database. Respondents were asked to indicate whether they worked with adults, children, or with both client groups. The term ‘child therapist’ was used throughout the survey to reflect those who work with children only (0‐15 years) and ‘adult therapist’ for those working with adults only (i.e. individuals of 16 years and above). Throughout this document the participants who work with both children and adults are referred to as ‘child and adult groups.’ Participants who reported working with both adults and children were directed, through the survey's branching design, to complete both the adult and child question sets. Their responses therefore appear in the tables as ‘mixed–adult’ and ‘mixed–child,’ reflecting answers provided from each clinical perspective. Because these responses relate to two distinct practice contexts, they are presented both separately, and collectively from the study's ‘mixed’ category; the term ‘mixed category’ is used consistently throughout the manuscript within tables and where appropriate, figures. A pilot study was conducted to assess clarity and relevance. Feedback from this process informed revisions to the survey and contributed to establishing face and content validity. Formal reliability analyses were not undertaken as part of this study. The term ‘written language differences’ is used as an inclusive descriptor encompassing a range of written language disorders and difficulties affecting writing and other written language processes (e.g., spelling and reading comprehension) across developmental and acquired contexts.

### 3.5 Data Analysis

Questionnaire responses were uploaded from JISC to SPSS data files (SPSS 28) for analysis. Analysis was conducted using the statistical software package R, including the packages haven, tidyverse, lme4, ordinal (Bates et al. 2015; R Core Team 2024; Wickham et al. 2023, Wickham et al. 2019).

Associations between adult and child groups’ responses were explored using ordinal regressions due to the non‐parametric nature of the data, with the ‘ordinal’ package in R (Christensen 2022; Liddell and Kruschke 2018). Although therapists working with children and adults were included in the analysis, for the purposes of this paper, results are primarily reported for the comparisons between the adult and child groups. An in‐depth analysis and interpretation of responses from therapists working with both children and adults is beyond the scope of this paper and will be reported elsewhere. Ordinal models were constructed with fixed effects of client group (adults or children), whether the respondent works with written language (yes or no), and the interaction of these two factors; and a random effect of respondent, except in pairwise comparisons, which had only fixed effects. All code used to generate inferential results and accompanying figures and tables are available at Open Science Framework (OSF) found at [OSF url: https://osf.io/snfd4/?view_only=4c7ae716a0b54be9a16ae32518da8136]. Qualitative responses were collated and examined for themes. The online survey allowed storage of all answers to the questionnaires, complete or otherwise. These were stored directly on a secure, university‐based server.

## 4 Results

The purpose of this study was to explore UK SLTs’ views on their role and scope of practice in written language assessment and intervention. Key findings of the study are reported below.

A total of 511 surveys were received. Table 1 summarises the demographic characteristics of the respondents. Respondents worked in a range of sectors and with a range of client groups spanning pre‐school children to adults. Employment sector referred to clinicians working within the public sector, the voluntary/charitable sector, the private sector, or as an independent practitioner. An independent practitioner refers to a speech and language therapist who is self‐employed and not employed by a public, private, or voluntary sector organisation. Respondents could tick all age bands that applied; therefore, percentages do not add up to 100%.

**TABLE 1:** Respondents' demographic characteristics.

Domain of interest	Answer	Number	Percentage (%)
*Professional status*	Speech and Language Therapist	461	90.2%
	Newly Qualified SLT Practitioner	27	5.3%
	Other^‡^	23	4.5%
*Employment sector*	Public Sector	398	77.9%
	Independent Practitioner^***^	50	9.8%
	Private Sector	39	7.6%
	Voluntary/Charitable org	24	4.7%
*Age range worked with*	Pre‐school	249	48.7%
	Primary	331	64.8%
	Secondary	283	55.4%
	Young Adults 16–25^**^	216	42.3%
	Adults 26–65	145	28.4%
	Adults 66+	119	23.3%
*Access CPD* ^*^ *in written language*	Yes	215	42.1%
	No	296	57.9%
*Pre‐registration training in links between language & literacy*	Yes	341	66.7%
	No	110	21.5%
	Don't know	60	11.7%

* CPD, Continuing Professional Development.

** It is challenging to categorise children 16 and older due to flexibility in the UK educational system. Our method used is described in the main text.

*** Independent practice indicates speech and language therapist not employed by an organisation.

^‡^ ‘Other’ refers to SLTs dually qualified or working in academic or research settings.

Respondents self‐identified as working with children (293), adults (118), or both (81); 19 did not specify. Of the 19 who did not respond to this question, 13 indicated they worked only with individuals aged 16+, two indicated they only worked with children under 16 and four indicated they worked with clients both above and below the age of 16. These 19 participants were ‘adult’, ‘child’, or ‘worked with both child and adult’ client groups. This resulted in 131 participants categorised as working with adults, 295 participants categorised as working with children, and 85 categorised as working with both adults and children.

Most respondents were SLTs working within the public sector (78%). The majority of SLTs worked with clients under 16. In addition, 67% of respondents reported receiving university training on links between language and literacy.

### 4.1 SLTs’ views Regarding Their Role in the Assessment of Written Language

**FIGURE 1:**
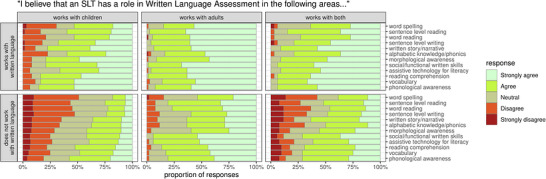
SLTs’ self‐rating of their role in written language assessment.

Table 2 shows SLTs’ views on their role in the assessment of written language. Respondents who work with adults agree significantly more strongly (Table 2 and Figure 1) that they have a role in assessing language differences across domains considered than those who work with children only (*β* = 2.51, SE = 0.54, *Z* = 4.68, *p* < 0.0001). In addition, we found that respondents who work on written language (Figure 1) agree more strongly that they have a role in written language than those who do not work on written language (*β* = 2.06, SE = 0.56, *Z* = 3.65, *p* = 0.0003). A post hoc pairwise comparison of the four subgroups of interest demonstrated that subgroups differ significantly, except in one case (See Table 3). Those working with adults only and not working with written language did not differ significantly from those working with children only and with written language. Across written language domains, the highest levels of agreement regarding SLTs’ perceived role in assessment were observed for phonological awareness (72%), reading comprehension (71%) and vocabulary (67%) (Table 2). In contrast, sentence level writing, word reading and word spelling were rated lowest overall. Despite agreement exceeding disagreement for sentence level writing (50% vs. 29%), word reading (49% vs. 28%), and word spelling (41% vs. 34%), these domains had the lowest levels of agreement regarding SLTs’ views on their role in written language assessment.

**TABLE 2:** SLTs’ views on their role in written language assessment.

Language domain	Strongly agree	Agree	Neutral	Disagree	Strongly disagree
Phonological awareness	181 (35.4%)	189 (37%)	84 (16.4%)	44 (8.6%)	13 (2.5%)
Reading comprehension	171 (33.5%)	193 (37.8%)	79 (15.5%)	48 (9.4%)	20 (3.9%)
Vocabulary	175 (34.2%)	168 (32.9%)	90 (17.6%)	61 (11.9%)	17 (3.3%)
Social/functional written skills	168 (32.9%)	173 (33.9%)	101 (19.8%)	51 (10%)	18 (3.5%)
Assistive technology for literacy	159 (31.1%)	161 (31.5%)	112 (21.9%)	59 (11.5%)	20 (3.9%)
Morphological awareness	129 (25.2%)	179 (35%)	119 (23.3%)	62 (12.1%)	22 (4.3%)
Written story/narrative	138 (27%)	164 (32.1%)	104 (20.4%)	79 (15.5%)	26 (5.1%)
Alphabetic knowledge/phonics	109 (21.3%)	166 (32.5%)	118 (23.1%)	94 (18.4%)	24 (4.7%)
Sentence level reading	115 (22.5%)	154 (30.1%)	112 (21.9%)	104 (20.4%)	26 (5.1%)
Sentence level writing	123 (24.1%)	132 (25.8%)	110 (21.5%)	115 (22.5%)	31 (6.1%)
Word reading	110 (21.5%)	138 (27%)	120 (23.5%)	115 (22.5%)	28 (5.5%)
Word spelling	97 (19%)	114 (22.3%)	129 (25.2%)	137 (26.8%)	34 (6.7%)

*Note*: Values represent the number of responses (percentage of total respondents) for each level of the rating scale across sub‐questions.

**TABLE 3:** Pairwise comparisons between subgroups defined by client group and whether they work on written language.

Client group (adult or child) contrasted with works with written language (yes or no)	Estimate (*β*)	SE	Z ratio	*p*‐value	Adjusted *p*‐value^*^
adult/yes—child/yes	2.51	0.53	4.68	<0.0001	<0.0001
adult/yes—adult/no	2.06	0.56	3.65	0.00026	0.0039
adult/yes—child/no	5.29	0.42	12.52	<0.0001	<0.0001
child/yes—adults/no	−0.44	0.58	−0.76	0.45	1
child/yes—child/no	2.79	0.44	6.33	<0.0001	<0.0001
adults/no—child/no	3.23	0.48	6.77	<0.0001	<0.0001

* Adjustments made using a Bonferroni correction for multiple comparisons.

### 4.2 SLTs’ views on Their Role in Direct Written Language Intervention

**FIGURE 2:**
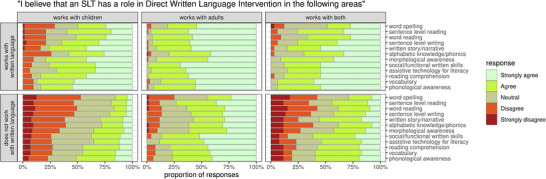
SLTs’ self‐rating of their role in direct written language intervention.

Table 4 shows respondents’ views on their role in direct written language intervention across the 12 written language domains. Similar to direct assessment, we found that respondents who work on written language agree significantly more strongly that they have a role in direct intervention than those who do not (*β* = 2.24, SE = 0.58, *Z* = 3.87, *p* < 0.0001). Furthermore, those who work with adults only agree more strongly than those who work with children only (*β* = 2.45, SE = 0.55, *Z* = 4.44, *p* < 0.0001) that they have a role in direct intervention. Post hoc pairwise comparisons (Table 5 and Figure 2) showed all relevant subgroups (those working with both child and adult client groups within or not within written language) differ significantly from each other, except in one case. Those working with adults only and not with written language did not differ significantly from those working with children only and with written language. Across domains, the highest levels of agreement regarding SLTs’ perceived role in written language intervention were observed for phonological awareness, reading comprehension and vocabulary. In contrast, word reading, word spelling and alphabetic knowledge/phonics were rated lowest overall. Despite this, agreement exceeded disagreement for word reading (43% vs. 31% disagree) and alphabetic knowledge/phonics (47% agree vs. 27% disagree), indicating that many SLTs still perceive a role for intervention in these areas. Views on word spelling were more evenly divided, with 36% agreeing and 36% disagreeing that SLTs have role in intervention.

**TABLE 4:** SLTs’ view on their role in written language intervention.

Language domains	Strongly agree	Agree	Neutral	Disagree	Strongly disagree
Phonological awareness	164 (32.1%)	183 (35.8%)	91 (17.8%)	55 (10.8%)	18 (3.5%)
Reading comprehension	154 (30.1%)	181 (35.4%)	91 (17.8%)	63 (12.3%)	22 (4.3%)
Vocabulary	164 (32.1%)	167 (32.7%)	96 (18.8%)	62 (12.1%)	22 (4.3%)
Social/functional written skills	159 (31.1%)	159 (31.1%)	118 (23.1%)	53 (10.4%)	22 (4.3%)
Assistive technology for literacy	152 (29.7%)	151 (29.5%)	131 (25.6%)	57 (11.2%)	20 (3.9%)
Morphological awareness	114 (22.3%)	173 (33.9%)	120 (23.5%)	77 (15.1%)	27 (5.3%)
Written story/narrative	135 (26.4%)	139 (27.2%)	117 (22.9%)	95 (18.6%)	25 (4.9%)
Sentence level reading	109 (21.3%)	135 (26.4%)	119 (23.3%)	117 (22.9%)	31 (6.1%)
Sentence level writing	116 (22.7%)	125 (24.5%)	119 (23.3%)	120 (23.5%)	31 (6.1%)
Alphabetic knowledge/phonics	101 (19.8%)	138 (27%)	135 (26.4%)	107 (20.9%)	30 (5.9%)
Word reading	103 (20.2%)	118 (23.1%)	130 (25.4%)	124 (24.3%)	36 (7%)
Word spelling	94 (18.4%)	91 (17.8%)	143 (28%)	142 (27.8%)	41 (8%)

*Note*: Values represent the number of responses (percentage of total respondents) for each level of the rating scale across sub‐questions.

**TABLE 5:** Pairwise comparisons between subgroups defined by client group and whether they work on written language.

Client group (adult or child) contrasted with works with written language (yes or no)	Estimate (*β*)	SE	Z ratio	*p*‐value	Adjusted *p*‐value^a^
Adults/yes—child/yes	2.45	0.55	4.44	<0.0001	0.00013
Adults/yes—adults/no	2.24	0.58	3.87	0.00011	0.0017
Adults/yes—child/no	5.59	0.43	12.88	<0.0001	<0.0001
Child/yes—adults/no	−0.21	0.60	−0.35	0.73	1
Child/yes—child/no	3.15	0.45	6.95	<0.0001	<0.0001
Adults/no—child/no	3.35	0.49	6.87	<0.0001	<0.0001

^a^ Adjustments made using a Bonferroni correction for multiple comparisons.

### 4.3 SLTs’ view on Their Role in *Indirect* Written Language Intervention

**FIGURE 3:**
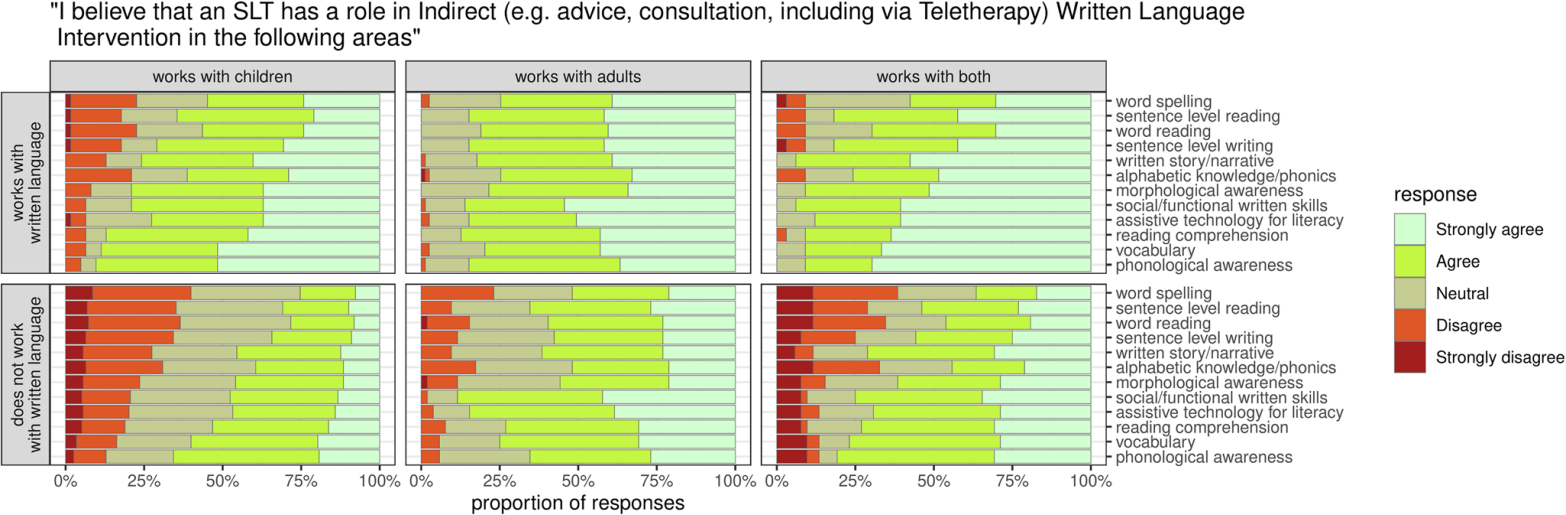
SLTs’ self‐rating on their roles in indirect written language intervention.

Table 6 shows respondents’ views on their role in indirect written language across the 12 written language domains at the level of indirect intervention. Consistent with findings across areas surveyed in this study, we found that respondents who work on written language agree more strongly that they have a role in indirect written language intervention (Figure 3) than those who do not work on written language (*β* = 1.72, SE = 0.64, *Z* = 2.7, *p* = 0.0100). However, the difference between respondents who work with adults only and those who work with children only did not reach the level of significance in this comparison (*β* = 1.18, SE = 0.61, *Z* = 1.95, *p* = 0.052). Post hoc pairwise comparisons (Table 7) showed fewer significant differences regarding this research question, but there was a striking pattern. Those who work with children only and not with written language differed significantly from all the other groups, but no other comparisons reached significance. Across domains, the highest levels of agreement regarding SLTs’ perceived role in indirect written language intervention were observed for phonological awareness, vocabulary and reading comprehension (Table 6). Although word reading, word spelling and sentence‑level writing were rated lowest overall in terms of SLTs’ perceived role in indirect written language intervention, agreement nevertheless exceeded disagreement across all three areas. Agreement was highest for sentence‑level writing (54% agree vs. 22% disagree), followed by word reading (48% vs. 25%) and word spelling (43% vs. 28%), although responses for word spelling indicated some uncertainty, reflected in a noticeable proportion of neutral responses (29%).

**TABLE 6:** SLTs’ views on their role in indirect written language intervention.

Language domain	Strongly agree	Agree	Neutral	Disagree	Strongly disagree
Phonological awareness	159 (31.1%)	223 (43.6%)	85 (16.6%)	33 (6.5%)	11 (2.2%)
Vocabulary	165 (32.3%)	202 (39.5%)	90 (17.6%)	41 (8%)	13 (2.5%)
Reading comprehension	151 (29.5%)	202 (39.5%)	100 (19.6%)	42 (8.2%)	16 (3.1%)
Social/functional written skills	157 (30.7%)	187 (36.6%)	108 (21.1%)	43 (8.4%)	16 (3.1%)
Assistive technology for literacy	151 (29.5%)	179 (35%)	119 (23.3%)	44 (8.6%)	18 (3.5%)
Written story/narrative	132 (25.8%)	186 (36.4%)	109 (21.3%)	68 (13.3%)	16 (3.1%)
Morphological awareness	120 (23.5%)	189 (37%)	128 (25%)	56 (11%)	18 (3.5%)
Sentence level writing	112 (21.9%)	165 (32.3%)	121 (23.7%)	92 (18%)	21 (4.1%)
Sentence level reading	109 (21.3%)	159 (31.1%)	127 (24.9%)	93 (18.2%)	23 (4.5%)
Alphabetic knowledge/phonics	109 (21.3%)	155 (30.3%)	131 (25.6%)	94 (18.4%)	22 (4.3%)
Word reading	98 (19.2%)	145 (28.4%)	140 (27.4%)	103 (20.2%)	25 (4.9%)
Word spelling	94 (18.4%)	123 (24.1%)	150 (29.4%)	116 (22.7%)	28 (5.5%)

*Note*: Values represent the number of responses (percentage of total respondents) for each level of the rating scale and sub‐questions.

**TABLE 7:** Pairwise comparisons between subgroups defined by client group and whether they work on written language.

Client group (adult or child) contrasted with works with written language (yes or no)	Estimate (*β*)	SE	Z ratio	*p*‐value	Adjusted *p*‐value^a^
Adults yes—child yes	1.18	0.61	1.94	0.052	0.78
Adults yes—adults no	1.72	0.64	2.70	0.0069	0.1
Adults yes—child no	3.93	0.47	8.35	<0.0001	<0.0001
Child yes—adults no	0.54	0.66	0.81	0.42	1
Child yes—child no	2.75	0.51	5.43	<0.0001	<0.0001
Adults no—child no	2.21	0.54	4.10	<0.0001	0.00063

^a^ Adjustments made using a Bonferroni correction for multiple comparisons.

### 4.4 SLTs’ Reported Involvement in the *Assessment* of Written Language Domains

The results show that SLTs reported involvement in the assessment of all written language domains (Table 8), although patterns of involvement varied by client group. Reported involvement was highest in domains where more than half of respondents indicated participation. SLTs working with adults only reported involvement across a range of written language domains with more than half of respondents indicating involvement in 9 of the 12 domains surveyed (58% to 89%). There were lower levels of reported involvement for phonological awareness, alphabetic knowledge and phonics and morphological awareness. In contrast, SLTs working with children only consistently reported lower levels of involvement across domains (ranging from 10%‐56%). Patterns of reported involvement differed by domain. Child only SLTs reported involvement in direct assessment for vocabulary and phonological awareness, whereas reported involvement was lower for literacy‐specific skills such as word reading (12%), sentence level reading (12%) and spelling (11%). Those who work with children and adults tend to report patterns of involvement in line with the developmental needs of the client group being considered. For example, mixed therapists reported greater involvement in vocabulary and phonological awareness when responding from a child‐focused perspective, but higher reported involvement in reading comprehension and vocabulary written language skills when responding from an adult‐focused perspective.

**TABLE 8:** SLTs’ reported involvement in Direct Assessment.

			Mixed	
Language domains	Child	Adult	Child	Adult
Vocabulary	144 (49.1%)	79 (66.9%)	51 (63%)	42 (51.9%)
Phonological awareness	163 (55.6%)	57 (48.3%)	52 (64.2%)	27 (33.3%)
Alphabetic knowledge/phonics	72 (24.6%)	31 (26.3%)	42 (51.9%)	19 (23.5%)
Word spelling	33 (11.3%)	75 (63.6%)	24 (29.6%)	23 (28.4%)
Word reading	36 (12.3%)	97 (82.2%)	26 (32.1%)	30 (37%)
Morphological awareness	92 (31.4%)	32 (27.1%)	40 (49.4%)	28 (34.6%)
Sentence level reading	34 (11.6%)	100 (84.7%)	30 (37%)	38 (46.9%)
Reading comprehension	81 (27.6%)	105 (89%)	42 (51.9%)	43 (53.1%)
Sentence level writing	36 (12.3%)	84 (71.2%)	23 (28.4%)	26 (32.1%)
Written story/narrative	63 (21.5%)	68 (57.6%)	41 (50.6%)	31 (38.3%)
Assistive technology	33 (11.3%)	83 (70.3%)	25 (30.9%)	26 (32.1%)
Social/functional application	29 (9.9%)	92 (78%)	25 (30.9%)	38 (46.9%)

*Note*: Values represent the rate of ‘Yes’ responses (% of client group) to the statement ‘I have a role in direct assessment’.

### 4.5 SLTs’ Reported Involvement in *Direct* Written Language Intervention

Information provided in Table 9 indicates that over 70% of those working with adults only reported providing direct written language intervention across 9 of the 12 domains, the exceptions being phonological awareness (54%), alphabetic knowledge/phonics (35%), and morphological awareness (33%). For those therapists working with both children and adults, over 50% reported involvement in direct intervention for adults across all domains except phonological awareness and alphabetic knowledge/phonics.

**TABLE 9:** SLTs’ reported involvement in direct intervention.

			Mixed	
Language domain	Child	Adult	Child	Adult
Vocabulary	35 (85.4%)	52 (72.2%)	20 (76.9%)	15 (57.7%)
Phonological awareness	30 (73.2%)	39 (54.2%)	19 (73.1%)	11 (42.3%)
Alphabetic knowledge/phonics	22 (53.7%)	25 (34.7%)	16 (61.5%)	9 (34.6%)
Word spelling	16 (39%)	58 (80.6%)	12 (46.2%)	13 (50%)
Word reading	19 (46.3%)	62 (86.1%)	14 (53.8%)	14 (53.8%)
Morphological awareness	26 (63.4%)	24 (33.3%)	16 (61.5%)	13 (50%)
Sentence level reading	23 (56.1%)	65 (90.3%)	14 (53.8%)	13 (50%)
Reading comprehension	31 (75.6%)	68 (94.4%)	18 (69.2%)	17 (65.4%)
Sentence level writing	23 (56.1%)	61 (84.7%)	16 (61.5%)	14 (53.8%)
Written story/narrative	28 (68.3%)	55 (76.4%)	19 (73.1%)	18 (69.2%)
Assistive technology	10 (24.4%)	55 (76.4%)	16 (61.5%)	16 (61.5%)
Social/functional application	16 (39%)	67 (93.1%)	12 (46.2%)	18 (69.2%)

*Note*: Values represent the rate of ‘Yes’ responses (percentage of client group) to the statement ‘I have a role in direct intervention’.

For those who work with children only, areas they reported the highest levels of involvement in direct intervention were vocabulary (85%), phonological awareness (73%) and reading comprehension (76%). The lowest rated domains for which they reported involvement was assistive technology for literacy (24%), word spelling (39%), word reading (46%). For those therapists working with both children and adults, over 50% of those reporting on the child‐focused practice of their work reported involvement in direct intervention for children across all domains except word spelling and social/functional application (46% respectively). For those working with both children and adults, reporting on the adult strand of their work, over 50% reported involvement in direct intervention in all domains except phonological awareness (42%) and alphabetic knowledge/phonics (35%).

### 4.6 SLTs’ Reported Involvement in *Indirect* Intervention Within Written Language Domains

Results as summarised in Table 10 indicate a high percentage of those who work with children reporting involvement in indirect intervention in the areas of phonological awareness 88%, vocabulary 85%, reading comprehension 72% and written story narrative 68%. There were higher percentage ratings for child only therapists and those working with both children and adults (child mixed therapists), regarding their reported involvement in indirect intervention for vocabulary (85% and 78%, respectively) and phonological awareness (88% and 75%, respectively). In addition, both groups had the lowest percentage ratings for reported involvement in word spelling (42%) and assistive technology (25% and 44%, respectively) and social/functional application (28% and 47%, respectively). For those working with adults only, there was more variability across domains regarding their reported involvement in indirect written language intervention. There was higher reported involvement for social functional application (81%), reading comprehension (77%), assistive technology (72%), and word reading (60%).

**TABLE 10:** SLTs’ reported involvement in Indirect Intervention.

			Mixed	
Language domain	Child	Adult	Child	Adult
Vocabulary	61 (84.7%)	26 (55.3%)	28 (77.8%)	22 (61.1%)
Phonological awareness	63 (87.5%)	17 (36.2%)	27 (75%)	15 (41.7%)
Alphabetic knowledge/phonics	36 (50%)	11 (23.4%)	22 (61.1%)	14 (38.9%)
Word spelling	30 (41.7%)	21 (44.7%)	15 (41.7%)	14 (38.9%)
Word reading	33 (45.8%)	28 (59.6%)	20 (55.6%)	16 (44.4%)
Morphological awareness	40 (55.6%)	14 (29.8%)	22 (61.1%)	17 (47.2%)
Sentence level reading	26 (36.1%)	27 (57.4%)	19 (52.8%)	16 (44.4%)
Reading comprehension	52 (72.2%)	36 (76.6%)	26 (72.2%)	25 (69.4%)
Sentence level writing	40 (55.6%)	26 (55.3%)	19 (52.8%)	14 (38.9%)
Written story/narrative	49 (68.1%)	24 (51.1%)	22 (61.1%)	20 (55.6%)
Assistive technology	18 (25%)	34 (72.3%)	16 (44.4%)	15 (41.7%)
Social/functional application	20 (27.8%)	38 (80.9%)	17 (47.2%)	20 (55.6%)

*Note*: Values represent rate of ‘Yes’ responses (percentage of client group) to the statement ’I have a role in Indirect Intervention’.

### 4.7 SLTs’ Views on Their Knowledge and Skills in the Written Language Domains

**FIGURE 4:**
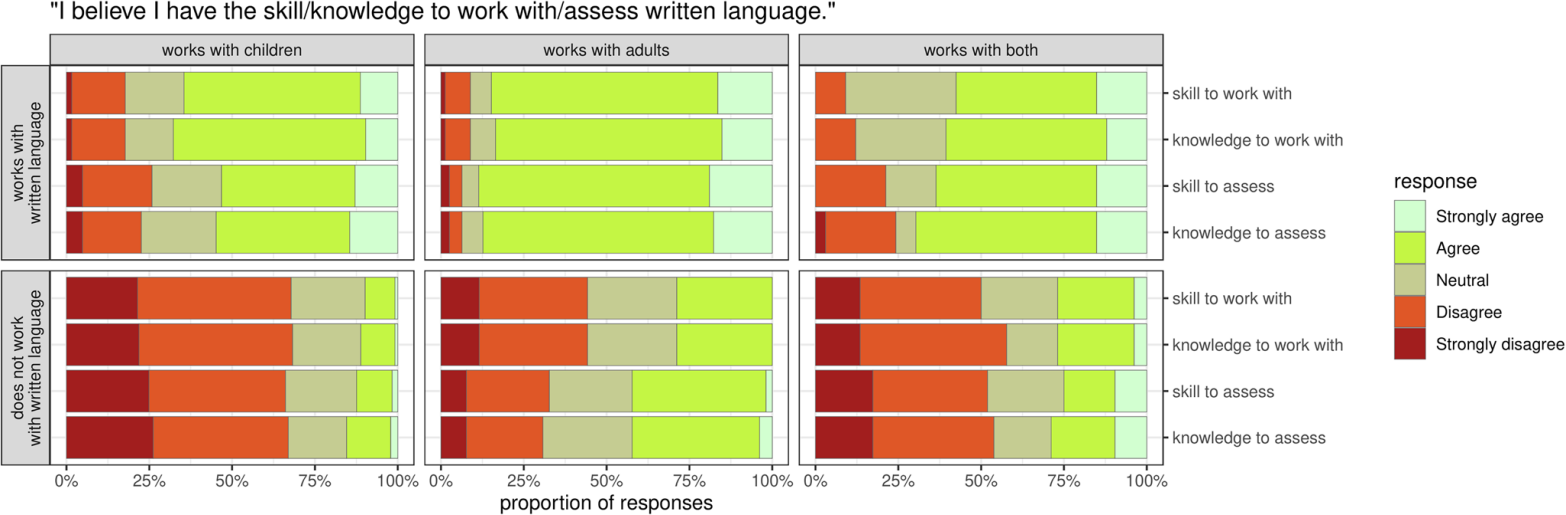
Self‐rated skills and knowledge for written‐language work.

Results as shown in Table 11 and Figure 4 indicate that those working with adults only are more likely to agree with each of the four statements below than those who work with children or those working with adults and children, regardless of whether they work on written language.

1. I believe I have the skills to work with written language.
2. I believe I have the skills to assess written language.
3. I believe I have the knowledge to work with written language.
4. I believe I have the knowledge to assess written language.

**TABLE 11:** SLTs’ self‐reported knowledge and skills for written language assessment and intervention.

I have the…	Strongly agree	Agree	Neutral	Disagree	Strongly disagree
knowledge to assess written language	40 (7.8%)	159 (31.1%)	85 (16.6%)	147 (28.8%)	80 (15.7%)
knowledge to work with written language	26 (5.1%)	157 (30.7%)	94 (18.4%)	168 (32.9%)	66 (12.9%)
Skills to assess written language	38 (7.4%)	150 (29.4%)	97 (19%)	150 (29.4%)	76 (14.9%)
skills to work with written language	29 (5.7%)	149 (29.2%)	105 (20.5%)	163 (31.9%)	65 (12.7%)

*Note*: Values represent the number of responses (percentage of total respondents) for each level of the rating scale.

A significantly higher proportion of therapists who work with adults only believe they have the knowledge (*β* = 1.04, SE = 0.33, *Z* = 3.21, *p* < 0.0001) and skills (*β* = 1.28, SE = 0.33, *Z* = 3.86, *p* < 0.0001) to assess written language compared to therapists who work with children. This pattern was also evident among SLTs working with adults, who reported greater perceived knowledge (*β* = 0.73, SE = 0.34, *Z* = 2.15, *p* = 0.03) and skills (*β* = 0.84, SE = 0.34, *Z* = 2.47, *p* = 0.01) in working within the written language domain, compared to those who worked solely with children.

Respondents who work on written language are more likely to strongly agree than those who do not work on written language, regarding their knowledge (*β* = 1.63, SE = 0.34, *Z* = 4.87, *p* < 0.0001) and skills (*β* = 1.83, SE = 0.34, *Z* = 5.35, *p* < 0.0001) to assess written language and their knowledge (*β* = 2.36, SE = 0.36, *Z* = 6.59, *p* < 0.0001) and skills (*β* = 2.43, SE = 0.36, *Z* = 6.79, *p* < 0.0001) to work on written language. There were no significant interactions in these comparisons. However, visual inspection and AIC (Akaike Information Criterion) comparison suggest that whether a respondent works with written language has a stronger influence on their responses than which client group they work with (See supplementary materials on OSF for details).

### 4.8 SLTs’ Views on What Their University Training Should Include

Many respondents surveyed reported that university training should include the links between spoken language and literacy, written language assessment and written language intervention.

**FIGURE 5:**
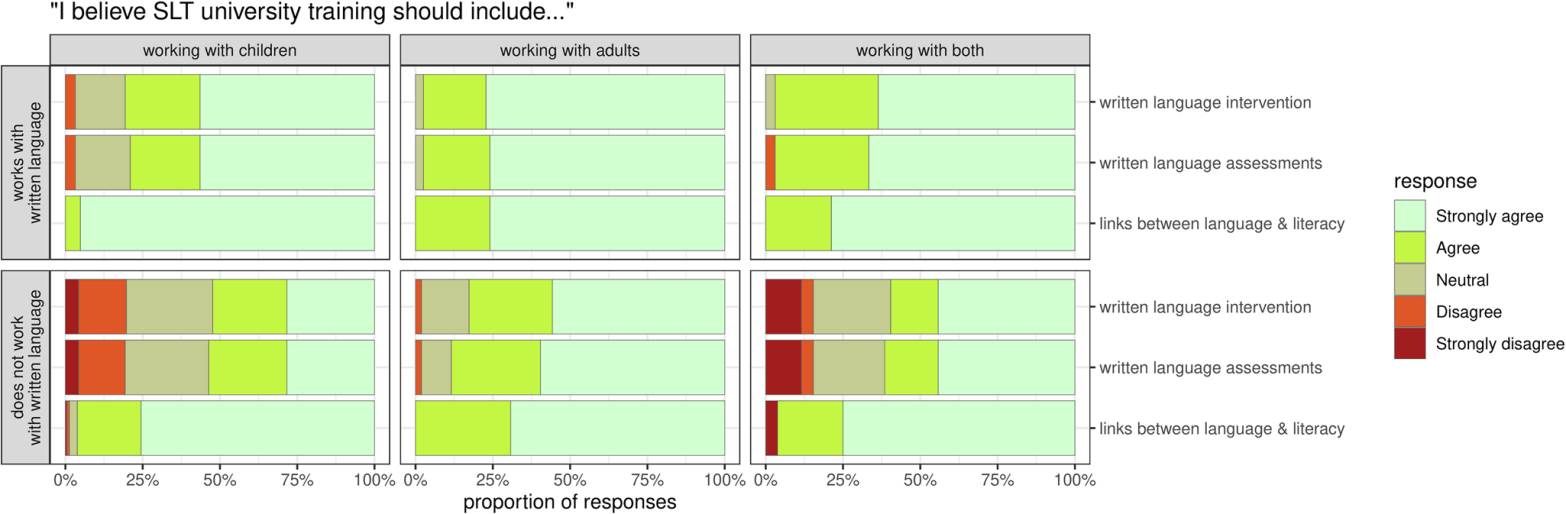
Ratings of statements of what university training should include.

A large majority of respondents (Table 12, Figure 5) agreed that university training should include links between language and literacy (98%), with lower but still substantial agreement for written language assessment (71%) and written language intervention (70%). Information within Table 12 and Figure 5 indicates a significant difference between respondents who work with adults only and those who work with children only (*β* = 1.12, SE = 0.54, *Z* = 2.09, *p* = 0.04), and between those working with written language and those not (*β* = 1.27, SE = 0.56, *Z* = 2.26, *p* = 0.02). Visual inspection, supported by pairwise comparisons (see supplementary materials), suggests that those who work with children and with written language agreed most strongly that training should include links between language and literacy. In contrast, those who work with children but do not work on written language disagreed more strongly that training should include written language assessment or intervention.

**TABLE 12:** SLTs’ views on written language content that should be included in university training.

University training should include:	Strongly agree	Agree	Neutral	Disagree	Strongly disagree
Links between language and literacy	396 (77.5%)	104 (20.4%)	6 (1.2%)	2 (0.4%)	3 (0.6%)
Written language assessment	237 (46.4%)	124 (24.3%)	93 (18.2%)	41 (8%)	16 (3.1%)
Written language intervention	235 (46%)	120 (23.5%)	99 (19.4%)	41 (8%)	16 (3.1%)

*Note*: Values represent the number of responses (% of total responses) for each level of the rating scale for sub‐questions.

### 4.9 SLTs’ Views on What Facilitates Their Working in Written Language Domains

**FIGURE 6:**
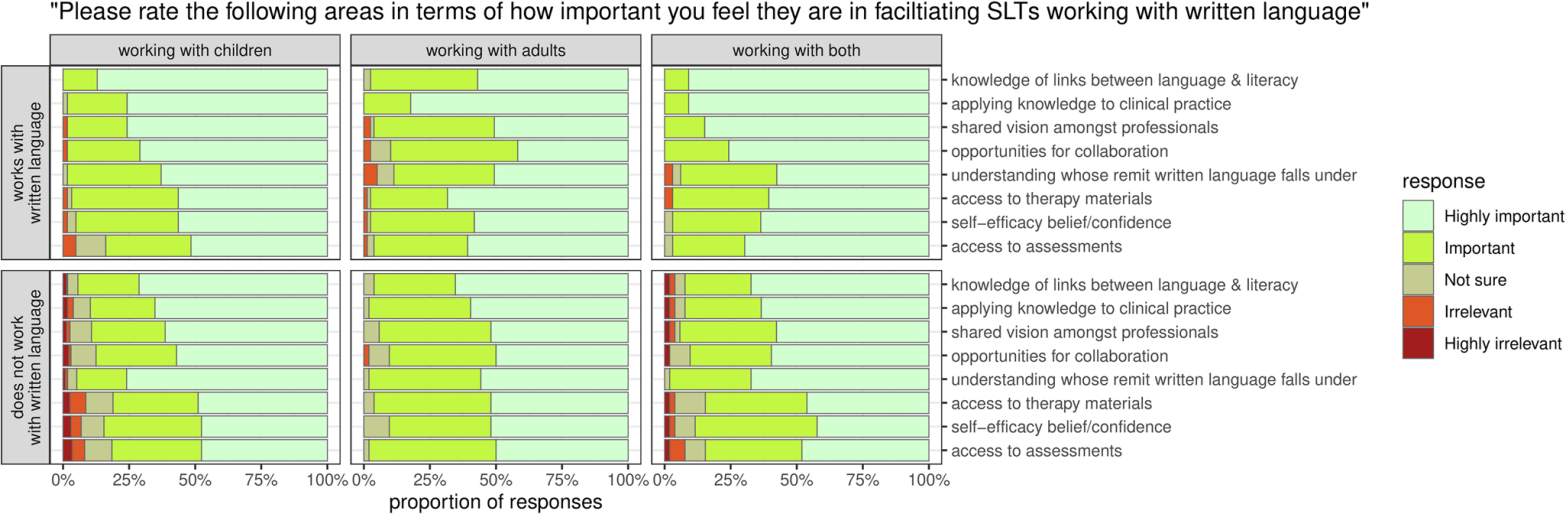
Importance ratings of factors facilitating SLTs’ written language practice by client group.

Over 50% of respondents surveyed (Table 13 and Figure 6) reported that all the domains listed are highly important regarding facilitating their work in written language. Knowledge of the links between language and literacy and applying knowledge to clinical practice were noticeably more highly rated (70% and 71%, respectively) than all other areas.

**TABLE 13:** SLTs’ view on facilitators to working on written language.

Domain considered	Highly important	Important	Not sure	Irrelevant	Highly irrelevant
Knowledge of links between language and literacy	364 (71.2%)	126 (24.7%)	15 (2.9%)	2 (0.4%)	4 (0.8%)
Understanding whose remit written language falls under	339 (66.3%)	146 (28.6%)	17 (3.3%)	7 (1.4%)	2 (0.4%)
Applying knowledge to clinical practice	358 (70.1%)	123 (24.1%)	19 (3.7%)	6 (1.2%)	5 (1%)
Shared vision amongst professionals	315 (61.6%)	161 (31.5%)	24 (4.7%)	7 (1.4%)	4 (0.8%)
Opportunities for collaboration	292 (57.1%)	171 (33.5%)	36 (7%)	6 (1.2%)	6 (1.2%)
Self‐efficacy belief/confidence	262 (51.3%)	196 (38.4%)	33 (6.5%)	12 (2.3%)	8 (1.6%)
Access to therapy materials	274 (53.6%)	178 (34.8%)	34 (6.7%)	18 (3.5%)	7 (1.4%)
Access to assessments	265 (51.9%)	180 (35.2%)	39 (7.6%)	18 (3.5%)	9 (1.8%)

*Note*: Values represent the number of responses (percentage of total responses) for each level of the rating scale for sub‐questions.

There was no detectable difference in views on those who work with adults only and those who work with children only regarding what facilitates their working in written language domains (*β* = ‐0.56, SE = 0.44, *Z* = ‐1.28, *p* = 0.20). There was also no detectable difference between those who work on written language and those who do not (*β* = 0.12, SE = 0.45, *Z* = 0.27, *p* = 0.79). Visual inspection of Figure 6, supported by pairwise comparisons (see Supporting Information), suggests that those who work with children and with written language feel overall that all areas listed are important.

### 4.10 SLTs’ Views on Whether the Profession Would Benefit From a Position Paper on Our Role in Written Language

Of 511 respondents, 453 (88.6%) felt that the profession would benefit from a position paper on the role of SLTs in written language. There were 9 (1.8%) respondents who felt this would not be of benefit and 49 (9.6%) did not know. Of the 9 respondents who felt that the profession would not benefit from a position paper (working with adults = 1, working with children = 7, working with both children and adults = 1), none reported working with written language, and none strongly agreed with having a role in any of the areas of written language surveyed.

In summary, the direction of the responses indicates that those who feel written language is not important to their role are least likely to agree that the profession would benefit from a position statement. Conversely, those who work in written language are more likely to support the need for a position statement.

Qualitative analysis of free text responses relating to what university training should include, the potential benefit of a position paper, and other comments provided clarity regarding the source of some of the observed patterns. Comments in favour of a position statement included the need for guidance: ‘*I feel like guidance on how to assess and manage written impairments would be beneficial’;* and ‘*… SLTs need to be clear why they might be involved in this type of assessment /intervention and what skills /resources they need to be equipped to do it’*. The following comments state how having guidance could help build confidence and help resolve areas of uncertainty: ‘*A position paper would help SLTs' confidence in written language practice’*; ‘*It would probably help to formalise the MDT [multidisciplinary team] roles in this area as there is overlap between OT [Occupational Therapist], SLT, education, visual impairment etc’*. Respondents also expressed the view that they feel it is within the adult therapists’ role ‘*literacy is an integral part of language skills which should fall under our remit, especially acquired dyslexia/dysgraphia/dyscalculia.’* However, there was acknowledgment of the implications for SLTs in taking on this domain of need, for example: ‘*are they* [SLTs] *the most appropriate person? if this is picked up by SLT—will this limit capacity in other key areas?’*

**FIGURE 7:**
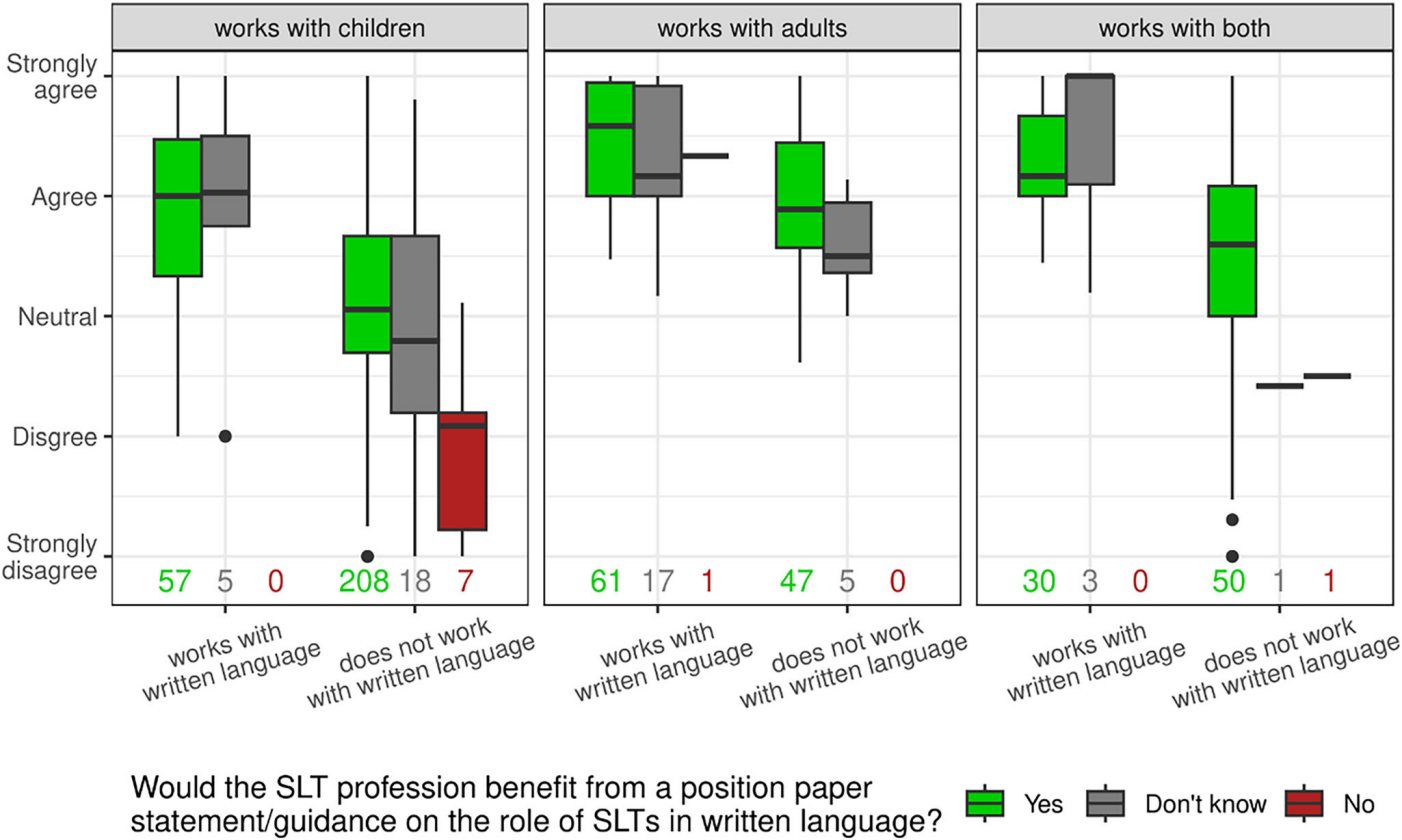
SLTs’ views on the need for a written language position paper by client group.

The data was further analysed to check whether there was an association between the group respondents work with (i.e. adults, children, adults and children), whether they work on written language or not and their views on the benefits of a position paper. The results (Figure 7) indicate that respondents are either in agreement for a position statement or ambivalent. Those who work with children only and work on written language tend to agree that the profession would benefit from a position statement. There was no consensus for those who do not work on written language. Those who work with adults only, whether they work on written language or not, generally agree that the profession would benefit from a position statement. Furthermore, therapists working with both children and adults who also work on written language tend to agree more that we would benefit from a position statement than those who do not work on written language.

In Figure 7, the vertical location of the boxes indicates where on the Likert scale therapists report viewing their roles in written language (as a combined score across the three areas). The colour indicates therapists’ opinions on whether a position paper would be beneficial, with the number of therapists contributing to each box listed along the bottom. Most surveyed therapists across all client groups agree that a position paper would be beneficial (indicated with green). However, those who work with children only and not with written language had more neutral views on their roles regarding written language (shown by median lines close to the ‛Neutral’ rating), as well as having more responses indicating they were not sure a position paper would be beneficial (N = 18) or disagreeing outright (N = 7).

### 4.11 The Association Between Client Group and Therapists Who Work on Written Language

**FIGURE 8:**
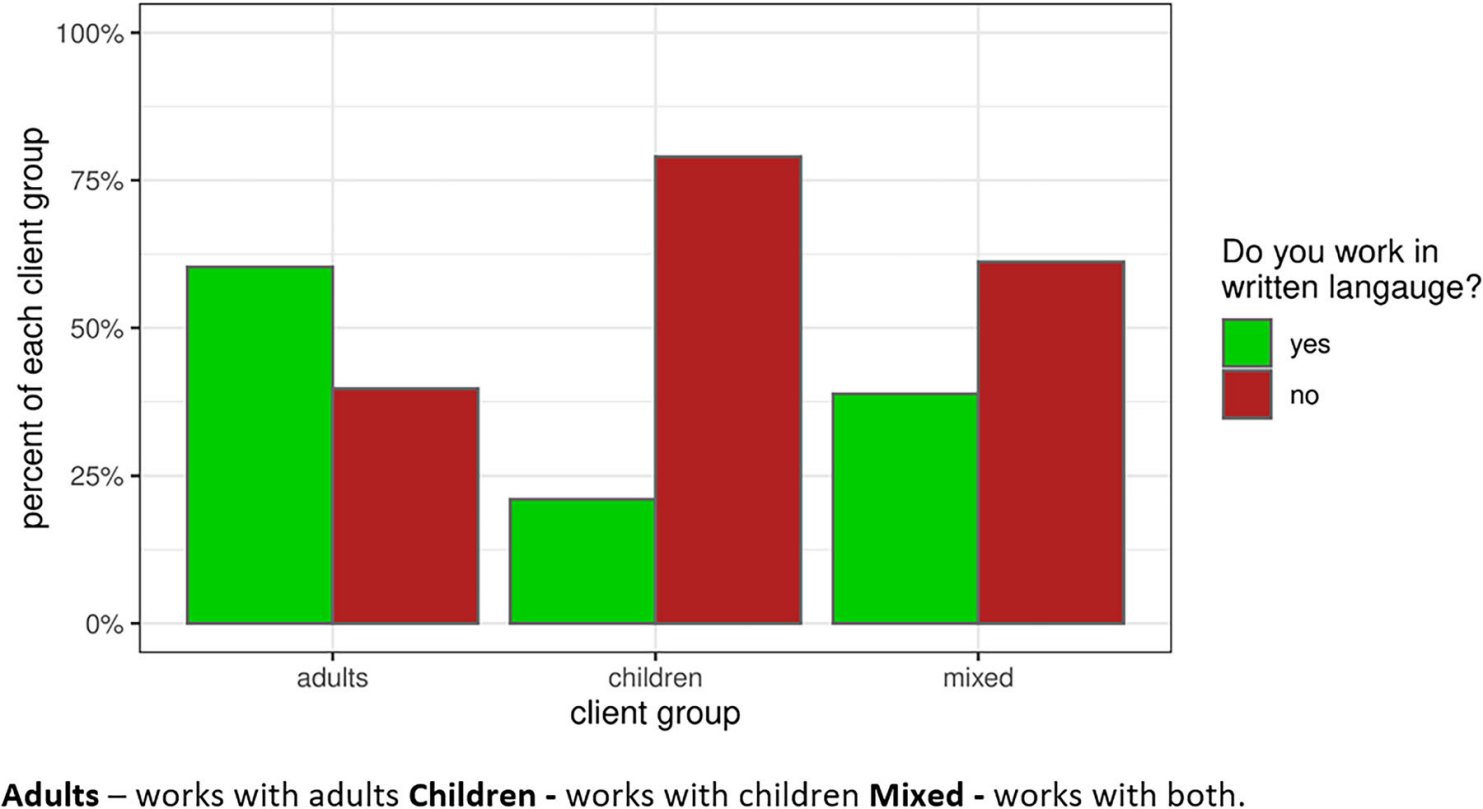
Association between client group and whether therapists work on written language.

There is a significant difference in how the respondents who work with adults only engage with written language as compared to those who work with children only and those who work with both adults and children (χ^2^ (2) = 63.39, *p* < 0.0001), where those who work with adults only are more likely than the other groups to work on written language. Figure 8 indicates that there is a significant difference in how the respondents who work with adults only engage with written language as compared to those who work with children only and those who work with both adults and children (χ^2^ (2) = 63.39, *p* < 0.0001), where those who work with adults are more likely than the other groups to work on written language.

## 5 Discussion

The purpose of this study was to explore UK SLTs’ views on their role and scope of practice in relation to written language, with a view to determining whether a formal position statement may be warranted. This discussion examines the key questions asked of participants and offers an interpretation of their responses in light of the overall of the study.

### 5.1 Do SLTs Have a Role in Direct and Indirect Written Language at the Level of Assessment and Intervention?

Findings from the survey suggest that most UK SLTs believe they have a role in direct and indirect written language assessment and intervention, aligning with international guidance (e.g., ASHA 2001; SPA 2011) and with research literature where the role of SLTs in literacy is well established for being key due to their training in linguistics (e.g., Ehren, 2002; Serry & Hammond, 2015; Snow 2020). Reported patterns of involvement broadly reflected these views, although important differences emerged according to client group and written language domains.

### 5.2 Direct Assessment of Written Language

Therapists who work with adults are statistically more likely to agree that they have a role in the assessment of written language, regardless of whether they work in this area. This difference may reflect established adult therapist practice in the UK, where written language is often addressed in the context of language differences and the need to draw on all potential modes of communication. In contrast, those who work with children were more likely to support a role in written assessments if they work in this area, suggesting one influences the other. Those who work with children reported lower levels of involvement in the assessment across the majority of domains.

Therapists working with both children and adults reported moderate agreement, suggesting that exposure to adult caseloads may broaden their views on their scope of practice in written language. This difference raises an important question about how age group influences perceived professional role in written language. In addition, it raises the question of whether those who work with children only should be encouraged to engage with written language, particularly given the overlap between spoken and written language differences in children and the implications for social, emotional, and educational development and progress.

Interestingly, those who work with adults only reported involvement in the assessment of a broad range of written language domains, with a lower percentage of therapists reporting involvement in alphabetic knowledge/phonics, morphological awareness and phonological awareness. In contrast, those who work with children reported lower levels (10‐31%) of involvement in fewer written language domains with only vocabulary and phonological awareness nearing or exceeding 50% (49% and 56% respectively). This may reflect differences in service expectations, available resources, local pathways, or perceptions of professional role, where spoken language is prioritised over written language within child services.

### 5.3 Direct Intervention in Written Language

Views around direct intervention in written language reflect the client's stage of life, specific areas of need (e.g., spelling, reading, written expression), and their broader profile of need, including the complexity, severity, and co‐occurrence of difficulties. Across client groups, and in line with the literature (Blood et al., 2020; Bridges and Kelley 2023; Serry & Levickis, 2020; Stephenson et al. 2024, 2025), UK SLTs report having a role in written intervention in areas with clear links to spoken language, including vocabulary, reading comprehension, and written story narrative. Therapists who work with adults also reported roles in assistive technology and social/functional applications of written language skills, such as texting and emailing, reflecting the practical needs of their client group. Those who work with children only tended to report involvement in language skills, such as vocabulary and phonological awareness, which are foundational for literacy. All client groups recognised the need to meet the functional needs of their clients. Therapists working with both children and adults tended to report a broader view on their role (working across more areas of written language), suggesting that working with adult clients may influence this and promote greater recognition of their role in written language work.

### 5.4 Indirect Intervention in Written Language

Patterns of reported roles in indirect intervention broadly mirrored those observed in direct intervention. SLTs who worked with written language were more likely to report a role in indirect intervention than those who did not work in this area. This aligns with prior research (e.g., Wolf et al. 2019) which highlights the role of language and listening comprehension in literacy development. Wolf et al.’s findings therefore underline the importance of language and listening comprehension as integral components of reading comprehension, reinforcing the view that SLTs are well‐positioned to support reading development, particularly when spoken language is compromised.

Reported involvement in indirect intervention appeared to reflect the developmental and functional priorities of the populations served. Child‐focused therapists reported greater involvement in foundational language and literacy skills such as vocabulary, phonological awareness and reading comprehension, whereas adult‐focused therapists reported greater involvement in functional literacy, assistive technology and social applications of written language. This pattern suggests that therapists adapt their written language practice to the communication demands and participation needs of their client groups.

Differences in views between those who work with adults only and those who work with children only highlight the need for professional guidance to help reconcile client needs, SLT expertise and perceptions of professional role in written language. Given the well‐established relationship between language and literacy, challenges in spoken language at any linguistic level are likely to affect written language development and functioning. The findings therefore raise important questions regarding the role of child‐focused SLTs in supporting written language. We suggest that SLTs have a role in the domain of written language across the lifespan, particularly for individuals who present with literacy related differences within the context of underlying language differences.

Across several domains, levels of agreement regarding professional role exceeded levels of reported involvement. This suggests that some SLTs may recognise written language as falling within their professional remit but encounter barriers to implementation in practice. Such barriers may include service expectations, confidence, role boundaries, training opportunities, workload pressures, or access to assessment and intervention resources. This interpretation is consistent with respondents’ views regarding facilitators of practice, where knowledge of language and literacy links, confidence, access to resources and opportunities for collaboration were all identified as important contributors to written language work.

### 5.5 How Do SLTs View Their Knowledge and Skills in Written Language?

SLTs’ views regarding their skills and knowledge are influenced more by whether they work on written language rather than the client group they work with. Again, those who work with adults only rate their skills and knowledge more highly than those working with children only. These differences could be partly attributed to SLTs who work with adults having more exposure to written language tasks as part of their typical practice in assessment and intervention. There appears to be considerably more written language assessment tools in routine use by UK SLTs working with adults than by those working with children. Adult SLT practice frequently includes assessments of reading and writing as standard components of communication assessment, particularly in services for aphasia and acquired neurological conditions (e.g., SLT UK Assessment Framework; Pearson Clinical UK assessment catalogue). In contrast, commonly used paediatric SLT assessments focus primarily on speech, language, and communication, with NHS and RCSLT paediatric assessment guidance emphasising spoken language rather than literacy specific assessment. This therefore supports the view that SLTs working with adults develop greater knowledge and experience in written language than those working with children only. Conversely, and consistent with the work of Farquharson (2015), those therapists who do not work on written language may feel a lack of ability due to limited experience and exposure.

### 5.6 What Should University Training Include?

In line with the findings of Loveall et al. (2022) and Watson et al. (2020), most respondents agree that university training should include the links between language and literacy, as well as written language assessment and intervention. This indicates a recognition among therapists of the importance of these aspects in their professional education and practice that is consistent with Serry and Levickis (2020) and Farquharson (2015) regarding the need for SLT training. However, recent research (Summy and Farquharson 2024) suggests that pre‐registration training alone may not be sufficient to prepare SLTs to work within the domain of written language. This, they suggest, is due to the challenge of translating theoretical knowledge into practice, particularly where understanding of the links between spoken and written language is superficial. SLTs may find it challenging to effectively apply their knowledge of language to support literacy skills such as reading, spelling and writing. This highlights a potential need for more collaborative working between educators and SLTs, where the expertise of each can be utilised to enhance practice and outcomes in literacy development.

### 5.7 What Do SLTs Feel Facilitates Their Working in Written Language Domains?

Across all therapist groups, the most highly rated facilitators for working in written language included knowledge of the links between language and literacy; applying knowledge to clinical practice; and understanding whose remit written language falls under. This finding aligns with the work of Serry and Levickis (2020). Evidently, UK SLTs surveyed perceive these elements as essential for effectively addressing written language challenges in clinical practice. Notably, on these issues there were no significant differences between those who work with adults only and those who work with children only or between those who work on written language and those who do not. The implication is that there is a shared understanding of what supports effective practice in this area. This focus on knowledge and scope aligns with professional expectations. The Health and Care Professions Council (HCPC 2023) highlights the importance of working within one's professional scope of practice, as well as drawing on appropriate knowledge and skills to inform clinical decisions. It is therefore not surprising that these aspects were highly rated by SLTs.

### 5.8 Do SLTs Feel We Would Benefit from a Position Paper/Guidance on Our Role in Written Difficulties?

Most respondents (89%) agreed that the profession would benefit from a position paper clarifying the role of SLTs in supporting written language. This suggests a widespread recognition of the need for formal guidance in this area. McLean et al. (2021) similarly highlighted the importance of clearer role definition in literacy related practice. They noted that SLTs and teachers often seek more explicit guidance regarding their respective contribution and role in literacy instruction. Support for a position paper was influenced by whether an SLT works with written language, rather than the client group they work with. As expected, UK therapists who do not view written language as relevant to their role were least likely to support the development of a position statement. Conversely, SLTs whose work involves written language were more likely to endorse the need for greater clarity through a position paper.

Qualitative findings further reinforce this need with respondents highlighting the importance of clearer guidance on assessment and intervention, increased support to develop confidence, and clarification of multidisciplinary roles. These views reflect the interdisciplinary nature of written language assessment and intervention, an issue also noted by McLean et al. (2021) who reported frequent overlap in responsibilities across professional groups. Such overlap is unsurprising given the shared contributions of educators, psychologists and SLTs to literacy‐related practice. A UK profession‐wide position paper could therefore serve as a valuable resource by articulating the scope of SLT involvement in written language and offering evidence informed guidance to clinicians. In summary, and in keeping with existing evidence (Stephenson et al. 2024), The findings suggest that SLTs who engage with written language are more likely to report confidence in their skills, a clearer understanding of their role and greater involvement in literacy related practice.

### 5.9 Limitations of Study

It is acknowledged that survey research has well documented limitations, particularly in relation to sampling, representativeness, and reliance on self‐report (Nelson and Gilbert 2021; Phillips et al. 2021). The limitations identified in this study are therefore consistent with those commonly encountered in survey‐based research in health professions education (Hochberg and Eakin 2024). To encourage honest and open responses, the survey was designed to be anonymous. While this approach is recognised as good practice for reducing social desirability bias and increasing response candour (Phillips et al. 2021), it meant that responses could not be linked to specific roles or levels of experience beyond what respondents chose to report. As a result, it was not possible to assess the full representativeness of the sample, and it remains unclear whether particular groups of UK SLTs (e.g., those with greater experience or heightened interest in written language) may have been over‐or under‐represented. This limits the extent to which the findings can be confidently applied to the wider profession.

In addition, participation was voluntary, introducing the potential for self‐selection bias, a recognised limitation of survey research (Nelson and Gilbert 2021). Respondents may have been particularly motivated to engage with the topic of written language or to influence practice in this area, which could have shaped the pattern of responses. The study also relied on self‐reported perceptions of professional roles rather than direct observation of practice, which may not fully reflect enacted clinical behaviour. Finally, while the questionnaire was piloted prior to dissemination, a more diverse panel of reviewers may have further strengthened the survey by incorporating a wider range of professional perspectives, as recommended in survey design literature (Hochberg and Eakin 2024). Despite these limitations, the survey included a large national sample of UK SLTs (n = 511), providing a broad overview of professional views; however, as participation was voluntary, the findings may not be fully representative of the wider profession. Nevertheless, the study provides valuable insight into current perceptions of SLTs’ roles in written language assessment and intervention. As such, the findings offer a robust foundation for informing discussion, future research, and potential professional guidance.

### 5.10 Future Directions

Future research could focus on three key areas. First, researchers could examine how therapists and other professionals collaborate to support written language development across the lifespan. Second, future studies could explore ways to address resource constraints that limit SLTs working with children from engaging in written language practice. Finally, research could investigate the factors that facilitate written language assessment and intervention in adult services and consider how these may inform practice development in children's services.

### 5.11 Implications for Professional Practice and Development

This study establishes UK SLTs’ views of their role in written language and their reported involvement in written language assessment and intervention. We have also argued that SLTs are well‐suited to contribute to the discussion of written language input given the links between spoken and written language. There is a place for SLTs to be in an advisory consultative role where they can draw on their knowledge and expertise in the links between language and literacy. However, previous research (Serry and Levickis 2020; Watson et al. 2020) indicates that SLTs are cautious about their role for several reasons. These include the belief that teachers hold primary responsibility for reading instruction and assessment, ability to identify areas of input for written language, and intervention approaches; this is despite SLTs naturally targeting linguistic subskills that underpin written language skills. Furthermore, many therapists feel they lack the necessary training in written language assessment and intervention techniques. In addition, there are concerns regarding workload and how a move to working on written language could negatively impact this. A counterargument is that written language support should be considered across the lifespan. If SLTs support written language in adult services, similar involvement earlier in development may be warranted, provided appropriate professional guidance and training are in place. Therefore, SLTs incorporating the links between spoken and written domains may provide a more holistic approach to intervention for these individuals. We can potentially address this by expanding SLTs’ pre‐registration training in written language across the lifespan and clarifying the profession's role. In the former, we can emphasise how spoken language skills underpin reading, writing, and spelling, building on the evidence that spoken language is the foundation of written language development (Sedita, 2023). This aligns with established and contemporary models of reading, including Scarborough's Reading Rope (Scarborough 2001), which illustrates the interdependence of language comprehension strands with word recognition; the Active View of Reading (Duke and Cartwright 2021), which extends this perspective by highlighting the bridging processes that connect spoken language to skilled reading; and more recently, the Reading Is Language model (Snowling and Hulme 2025) which emphasises reading as a language based skill developing continuously across the lifespan. In practice, equipping SLTs to target spoken language is likely to benefit written language outcomes across reading, spelling and writing and helps to operationalise the scope of SLT involvement in written language within interprofessional teams. The current study underscores the profession's support for clearer guidance and training focus, suggesting that explicit attention to spoken language and literacy links during pre‐registration could strengthen confidence, competence, and uptake of written language engagement among SLTs across the lifespan. The implication is that clarifying SLTs’ role in written language will require targeted education on how intervention in language domains such as phonological awareness, vocabulary, grammar, narrative structure, and morphological awareness can support and facilitate written language development.

If we are advocating that SLTs do have a role in written language, an important question is whether their involvement should be direct (e.g., face‐to‐face intervention or assessment), indirect (e.g., advice, training, or consultation), or a blend of both. The balance between direct and indirect input is unlikely to be binary and may be shaped by clients’ needs, service setting, available resources, and the individual SLTs’ training and confidence (Bridges and Kelley 2023; Serry and Levickis 2020).

These findings support the view that SLT involvement does not always need to be direct and may be effectively delivered through indirect approaches. For example, over 20 years ago Staskowski and Zagaiski (2003) suggested that SLTs can contribute to and support children's written language development by building strong collegial team relationships. The suggestion here is that through collaborative practices between SLTs and educators, children's literacy development and progress can be enhanced. This emphasis on collaborative practice is also reflected in recent SEND policy, which advocates for joint working across education and health to support children's learning needs (Department for Education 2025/2026). To effectively support and engage with individuals with written language differences, SLTs must be equipped to assess the subsystems of language that are key for literacy (phonology, morphology, semantics, syntax, pragmatics) and to identify when differences in one or more of these areas may place individuals at risk of not just spoken language but also written language challenges.

If we accept that SLTs have a role in written language across the lifespan, then we need to prepare pre‐registration SLTs to collaborate in a meaningful way with educators to support teaching and learning needs of children with literacy needs that sit within the context of speech, language, or communication needs. The level and degree of interprofessional communication (IPC) will be influenced by how much others understand the role of SLTs (Fallon and Katz 2011). Understanding of each other's role would be supported through interprofessional education (IPE) and education‐based placements. Together these would provide opportunities for students from different professions to learn with, from, and about each other. IPE would help support mutual understanding of and respect for each other's role and expertise. For practicing SLTs, continuing professional development on the links between language and literacy and how to apply to practice would support their professional practice. Given this and building on the previous suggestions of Staskowski and Zagaiski (2003) together with the more recent work of McLean et al. (2021) and Stephenson et al. (2024), it seems clear that effective IPC between SLTs and educators requires a shared understanding of each profession's role. Teachers often lack awareness of the depth and breadth of SLTs’ scope of practice and how their expertise in language can inform literacy progress and development (McLean et al. 2021; Stephenson et al. 2024). This lack of knowledge can mean that opportunities for IPC can be missed. To bridge this gap, joint professional training is key where educators can deepen their knowledge of language and links to literacy and SLTs can enhance literacy assessment and intervention practices by aligning speech, language, and/or communication goals with classroom objectives. However, there are important caveats to bear in mind here: need for supportive school structures that can facilitate co‐planning and teaching; agreement regarding roles and responsibilities; and due regard to barriers such as time and workforce capacity. Interestingly, the RCSLT (2021) does recognise and acknowledge the role of SLTs in written language, as it positions them as essential contributors through their expertise in spoken language, implying their integral involvement in specialist provisions and intervention strategies that support literacy development. Further, the Health and Care Professions Council (HCPC 2023), the statutory regulator for SLTs in the UK, states in the Standard of Proficiency (SoPs) 12.6 that *SLTs must understand educational theory and the relationship between language and literacy, including sound‐awareness and school readiness*. This reflects HCPC's expectation that SLTs integrate educational and therapeutic knowledge to support children's literacy development and collaborate with educators to promote early learning outcomes.

To prepare SLTs to effectively engage with written language across the lifespan, university programmes should include introductory training that highlights the link between spoken and written language. Building on this foundation, SLTs can then choose to specialise further in literacy related practice. As Blood et al. (2010) suggest, this would require enhanced education, clinical training, and access to appropriate resources and support systems to ensure therapists are equipped to deliver effective written language output. A caveat to this is that SLTs’ capacity to engage in literacy related work is influenced not only by training (Stephenson et al. 2025) and role clarity, but also by systemic pressures within the profession. For example, since 2020 the impact of COVID‐19 has contributed to increased caseloads (Brito‐Marcelino et al. 2020), intensifying workload management challenges and raising concerns around mental health and wellbeing. Added to this is the ongoing shortage of SLTs (RCSLT, 2025), which places further strain on services and contributes to long waiting lists. These resource constraints must be acknowledged when considering how SLTs can be supported to expand their role in written language assessment and intervention.

Our final point concerns the need for a position statement. We argue that a formal statement from the RCSLT would strengthen the UK SLT profession across work with both children and adults. This would provide clarity and legitimacy for pre‐registration training, as well as guidance for post‐registration practice in relation to SLTs’ roles in written language across the lifespan.

## 6 Conclusion

This study explored UK SLTs’ views on their role in written language across the lifespan, alongside their reported involvement in written language assessment and intervention. Given the close relationship between spoken and written language, and SLTs’ expertise in core linguistic domains, they are well placed to contribute meaningfully to written language through direct intervention, collaborative practice, and support for educators and learners with written language differences.

Decisions about whether SLTs engage in written language intervention with children are shaped by a combination of internal (within clinician) and external (contextual) factors. Internal factors include pre‐ and post‐registration training (Blood et al. 2010; Fallon and Katz 2011), their confidence in addressing written language needs (Fallon and Katz 2011; Yi and Ericksen 2024), and their beliefs about professional roles (Loveall et al. 2022; Watson et al. 2020). External factors include wider service level policies including departmental policies, collaborative opportunities, available resources (Stephenson et al. 2024), and the lack of clarity regarding scope of practice in written language work (Loveall et al. 2022). Together, these factors can constrain SLTs’ involvement in written language support within educational contexts.

In contrast, therapists working with adults appear to have greater clarity regarding their role in written language, particularly in relation to acquired communication impairments, such as aphasia. Within the field, written language is routinely embedded within assessment and intervention, supported by well‐established tools and clinical guidance (e.g., Comprehensive Aphasia Test‐Swinburn et al., 2023; Comprehensive Assessment of Reading in Aphasia‐Webster et al. 2018; and Whitworth et al. 2014). This reflects a more clearly articulated professional remit and longer established evidence base within adult services.

There is therefore a clear need for greater consistency and clarity regarding UK SLTs’ roles in written language across the lifespan. The findings of this study suggest that a profession‐wide position statement is well supported allowing for more coherent practice, informing education and training, and strengthening UK SLTs’ contribution to written language with both children and adults.

## Funding

The authors received no specific funding for this work.

## Ethics Statement

This research received ethical approval from Newcastle University.

## Consent

By taking part in this survey, all participants consented to the study. No material from other sources was produced in this research.

## Conflicts of Interest

The authors declare no conflicts of interest.

## Data Availability

The data reported in this paper are available from the authors.
